# Accuracy Analysis of Computer-Assisted and Guided Dental Implantology by Comparing 3D Planning Data and Actual Implant Placement in a Mandibular Training Model: A Monocentric Comparison between Dental Students and Trained Implantologists

**DOI:** 10.3390/jpm13071037

**Published:** 2023-06-24

**Authors:** Marcel Ebeling, Andreas Sakkas, Alexander Schramm, Frank Wilde, Mario Scheurer, Karsten Winter, Sebastian Pietzka

**Affiliations:** 1 Department of Cranio-Maxillo-Facial-Surgery, German Armed Forces Hospital Ulm, 89081 Ulm, Germany; 2 Department of Cranio-Maxillo-Facial-Surgery, University Hospital Ulm, 89081 Ulm, Germany; 3 Institute of Anatomy, Medical Faculty, University of Leipzig, 04109 Leipzig, Germany

**Keywords:** dental implant planning, surgical guides, surgical templates, computer-assisted surgery, computer-aided surgery, guided implant surgery

## Abstract

The aim of this study was to investigate how precisely implantation can be realized by participants on a phantom head according to preliminary planning. Of particular interest here was the influence of participants’ previous knowledge and surgical experience on the precision of the implant placement. The placed implants were scanned using an intraoral scanner, saved as STL files, and superimposed with the 3D-planned implant placement. Deviations from the planning were indicated in millimeters and degrees. We were able to show that on average, the deviations from computer-assisted 3D planning were less than 1 mm for implantologists, and the students also did not deviate more than 1.78 mm on average from 3D planning. This study shows that guided implantology provides predictable and reproducible results in dental implantology. Incorrect positioning, injuries to anatomical structures, and implant positions that cannot be prosthetically restored can thus be avoided.

## 1. Introduction

Three-dimensional-planned implantology is a widely approved procedure in dental rehabilitation today. Even though experienced implantologists still insert a large number of implants freehand, the 3D-planned procedure appears to be an optimal solution, both in difficult situations and in the context of training. In its early days, implantation, and especially transfer to the surgical site, were challenging and only indicated in special situations. In the beginning, this method was only used at larger centres and special clinics [1]. Initially, the generation of 3D radiographs alone posed a challenge. At that time, CT was the only option for high-resolution 3D bone imaging. This was operated by radiologists and was only available to a limited extent in dental care. With the development of cone beam CTs, these 3D images became increasingly available for implantology [2,3]. Over time, different software solutions for implant planning were offered. Some of these were linked to individual implant manufacturers and others enabled planning with various systems from different manufacturers [4]. Approaches to transferring the implant position to the operating situs varied from different navigation systems to drilling templates and finally to robot-assisted surgery [5,6,7,8]. The further development of optical scanners and 3D printers enabled the further digitalisation of planning, as well as the production of guiding templates [9,10,11,12]. The use of biocompatible resins in the 3D printing process expands the spectrum of surgical applicability [13]. The safe clinical applicability of template-guided 3D-planned implantology could also be shown in larger patient collectives [5]. For other methods, the uncomplicated clinical applicability, as well as the precise transfer into the surgical site, must still be proven.

The exact and prosthetically useful positioning of implants is one of the decisive factors that has a direct impact on the success of implant treatment and implant survival [14,15]. The implantologist is still mainly responsible for the prosthetic positioning and the possibility of a later prosthetic restoration of the implants. But especially in the digital workflow of 3D planning and the production of drilling templates by a dental technician, special features can already be considered and possibly positively influenced.

Backward planning, considering the later prosthetic position by using a referencing template during 3D imaging or subsequently by using a digital wax-up in the planning software, can have further positive effects on the quality of implantological dental rehabilitation [16,17,18,19]. In surgery, as in implantology, experience and the development of personal surgical skills are of particular importance. Learning curves are indisputable [7,20,21,22,23]. A legitimate goal here is certainly to minimize intraoperative risks and aim for maximum success through thorough surgical preparation and planning [24]. This seems to be another advantage of 3D implant planning and guided implantology. During the training of oral and maxillofacial surgeons, training assistants can discuss the implant planning with their teachers at a distance from the patient and without time stress. The fact that a high level of intraoperative precision can be achieved through 3D planning, even by doctors in training, has already been demonstrated in other areas of maxillofacial surgery, such as orthognathic surgery [25,26]. Therefore, the use of computer-assisted surgical planning seems to be a very useful element, especially during student training [27,28,29]. Particularly, in implantology, surgical and prosthetic elements, but also the special features of dental technology, can be taught and learned in this way. 

Therefore, courses in 3D-planned and guided implantology are regularly offered at the Department of Oral and Maxillo-Facial Surgery of the University of Ulm for undergraduates, as well as for fully licensed dentists. During this training, the students and implantologists independently plan implants on the computer according to instructions. Furthermore, all participants insert an implant according to a preexisting plan using a 3D printed drilling template in a CAD (computer-aided design)/CAM (computer-aided manufacturing) mandibular model of a phantom head.

The aim of this study was to investigate how precisely the implantation can be realized by participants on the phantom head according to the preliminary planning. Of particular interest here was the influence of participants’ previous knowledge and surgical experience on the precision of the implant placement. Therefore, the results of students placing an implant for the first time should be compared with those of experienced implantologists (IP).

## 2. Materials and Methods

As part of a dental implantology workshop at the Department of Oral and Maxillo-Facial Surgery at the university hospital Ulm, 40 DSs from various semesters and 20 either self-trained or certified IPs were included in this study.

All 60 participants of the course received the same patient case already planned with coDiagnostiX™ 9 (Dental Wings GmbH, Berlin, Germany) by the Head of the oral and maxillo-facial surgery department of the university hospital Ulm, Germany, for implantation of the right and left first molars. A corresponding CAD/CAM-produced drilling template/surgical guide was used, and a corresponding mandibular model was handed out to the participants. 

In this completely digital workflow, the patient is first scanned with a 3D CT-/CBCT scan available on the market (KaVo 3D Exam, KaVo Dental GmbH, Biberach an der Riß, Germany, was used). A scan template is not necessary. The patient’s mouth is then scanned with an intraoral scanner. Alternatively, a surface scan of the master model or an impression scan can be used. Such a scan is necessary to ensure the exact fit of the digital surgical guide. The CT/CBCT scan (DICOM data) and the intraoral/surface/impression scan (STL file) are imported into coDiagnostiX™ and matched. In partially edentulous cases and edentulous cases, residual dentition and temporary implants can be used for matching, respectively. Then, the planning of the implant is personalized, considering the patient’s anatomy and the desired prosthetic result. Once the planning process is complete, the digital drill guide must be designed and exported as an STL file. The STL file of the surgical guide designed in coDiagnostiX™ is then sent to a calibrated 3D manufacturing system, and is CAD/CAM-fabricated. The drill template is produced, and the sleeves are finally inserted. There are different ways to produce drilling guides with 3D production systems (e.g., 3D printer or milling machines). The prerequisite for this is that the respective manufacturing system can read STL files and process biocompatible material.

The actual implantation on the patient and consecutive prosthetic restoration were followed for the “real” patient. The corresponding mandibular model was specifically CAD/CAM-produced for the workshop.

**Figure 1:**
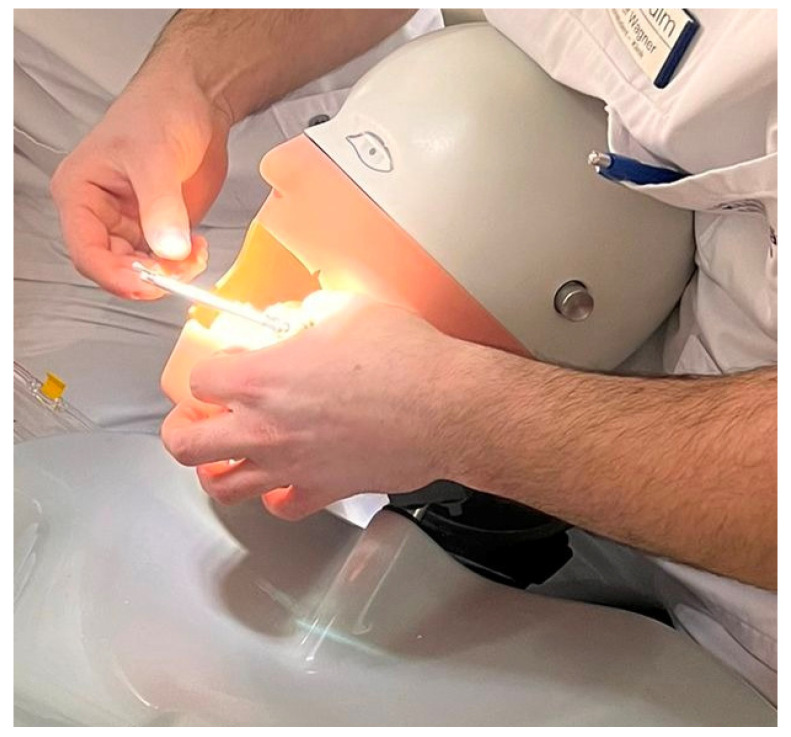
Dental implantation on the mandibular model on a phantom head.

Subsequently, step-by-step instructions were given by the Head of the oral and maxillo-facial surgery department of the university hospital Ulm, Germany, and the implantation of the left first molar was carried out step-by-step (Figure 1).

**Figure 2:**
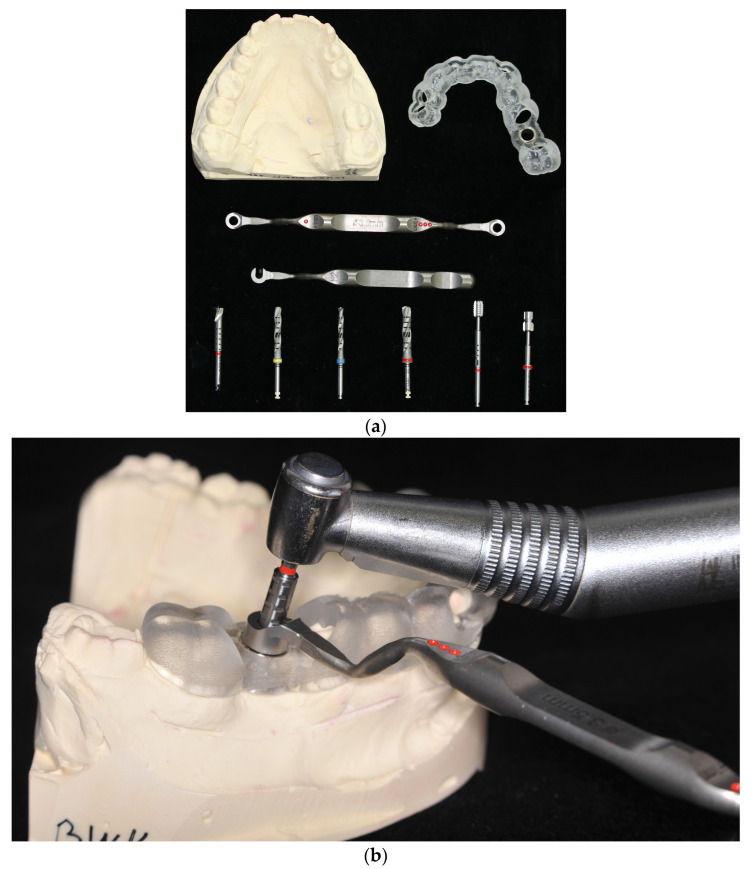
**(a)** Overview of all surgical drills and the drilling guides used, (**b**) and using one surgical drill with the drilling guide.

The implantologists implanted both teeth in one model, while the students shared the model and implanted only one of the two teeth. This was followed by the implantation of the right first molar without further step-by-step instructions (Figure 2).

**Figure 3:**
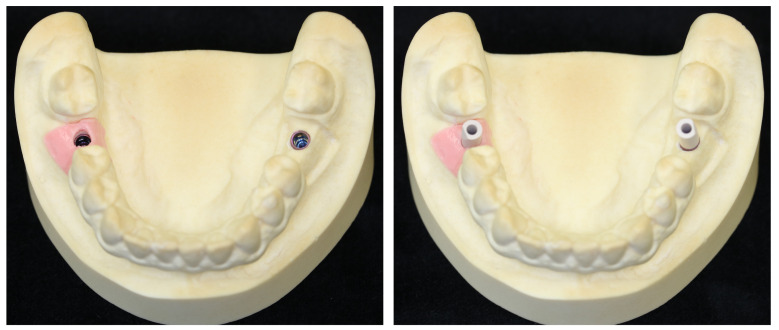
Mandibular model with a gingival mask (right molar) and removed gingival mask (left molar) (**right**), and with the scanbody screwed on in the region of the first molars (**left**).

**Figure 4:**
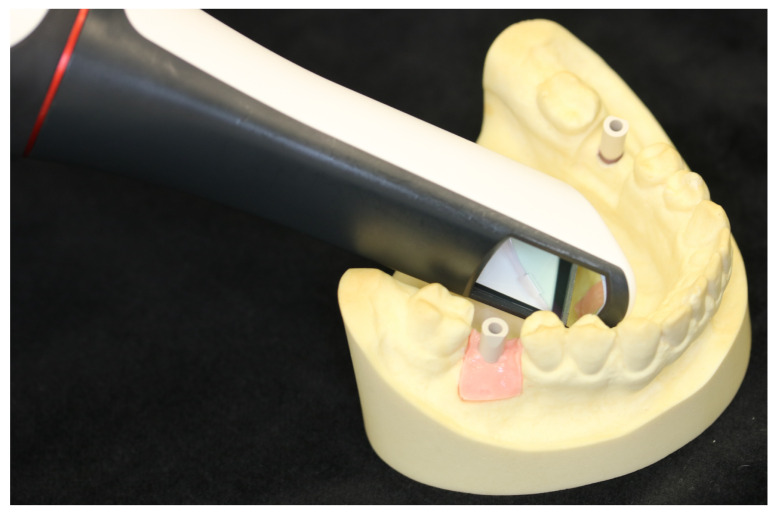
Digital impression of the inserted implant using the Trios 4 (3 Shape, Denmark) intraoral scanner.

After the implantation was completed, the implant position was recorded by applied scan bodies and an intraoral scan (Trios 4, 3 Shape, Copenhagen, Denmark, see Figure 3 and Figure 4). 

**Figure 5:**
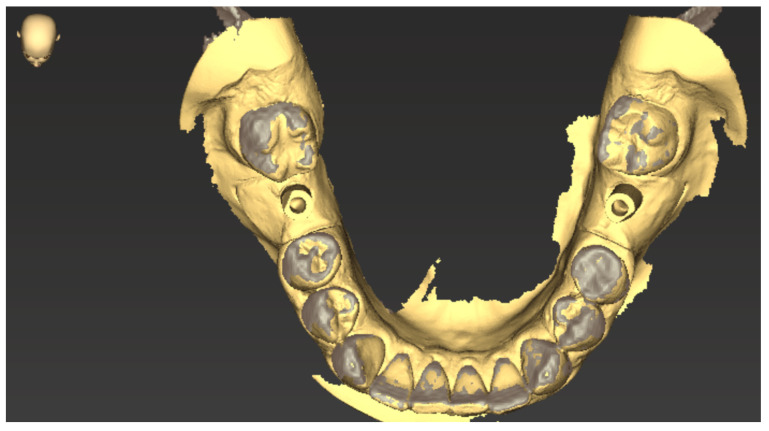
Superimposed initial planning of the implants with the actual implant position after scanning.

The scan data were saved as an STL file and could be reloaded into coDiagnostiX™ 9 in a further step. Using the software “treatment evaluation” function, the initial 3D planning of the implants could be superimposed with the actual implant position after model scanning (Figure 5).

**Figure 6:**
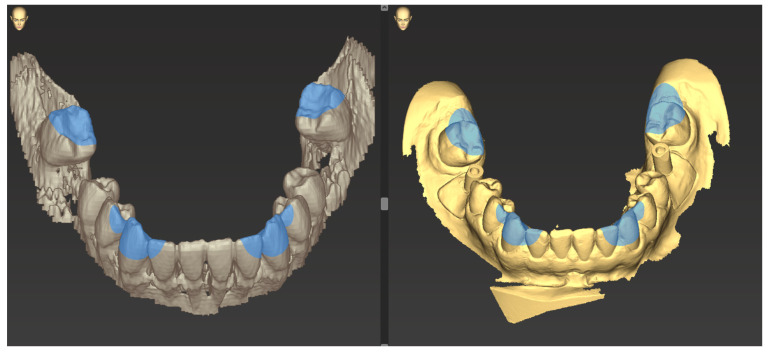
Selecting the canines and mesio-buccal cusp tips as the reference markers for superimposition in the planning scan (**right**) and the mandibular model scan (**left**).

The chewing tip of the two canines and the mesio-buccal cusp tip of the first molar were used as non-variable matching zones for matching the two scans (Figure 6).

**Figure 7:**
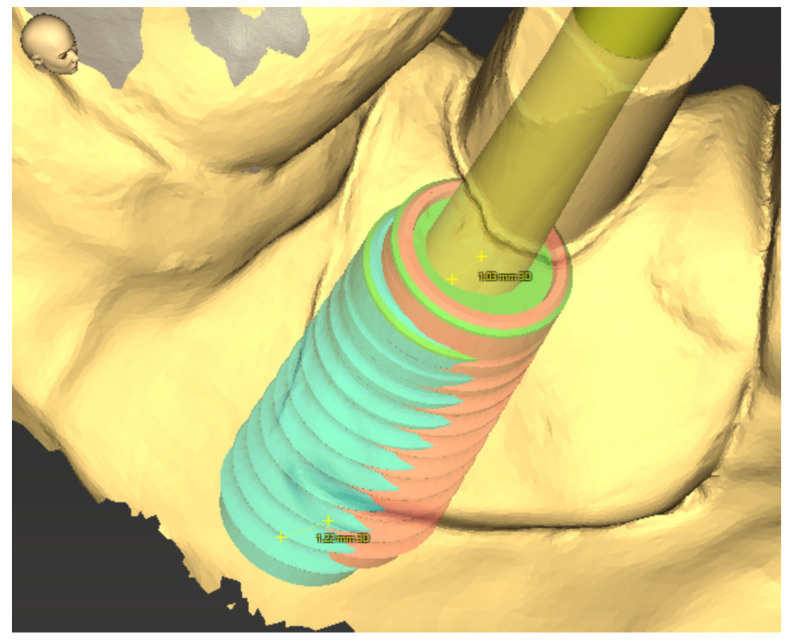
Three-dimensional imaging of the calculated 3D offset of the actual implant position of 1.03 mm at the implant tip and 1.22 m at the implant base.

The software then calculates the deviation of the implant angle in degrees, as well as the 3D offset, the distal/X-axis, vestibular/Y-axis, and apical/Z-axis deviation for the implant base, and analogously for the implant tip in millimeters (Figure 7). A negative value for these parameters implies an implant placement too deep, too distal or too vestibular.

The results of the analysis were anonymously transferred to a Microsoft Excel (Version 16.74, Microsoft®, Redmond, WA, USA) spreadsheet and analyzed descriptively.

To compare continuous variables among the groups, first the normality assumption was tested using the Shapiro–Wilk test. If the normality assumptions were met for a given continuous variable, the Mann–Whitney test (with or without the equality of variances assumed) was applied to compare the distribution of this variable among the groups of patients. We interpreted only the results significant at the level of 0.05. All calculations were conducted using R studio ver. 2022.07.1., Posit PBS, Vienna, Austria.

As this is a purely experimental study without patient participation, ethical approval for this study was not needed. This research was conducted in full accordance with the ethical standards of the institutional research committee, as well as the 1964 Helsinki Declaration and its later amendments or comparable ethical standards.

## 3. Results

### 3.1. Comparing Dental Students and Implantologists to 3D Planning

In the group of implantologists, we found that the parameter of the implant angle, 3D offset, the distal, vestibular and apical deviation of the implant base and analogously for the implant tip in millimeters, on average, deviated less than 1 mm from the actual planning. The implantologist implants deviated with a mean value of 2.13 mm from the planned implant placement. A mean deviation of 0.74 mm for the 3D offset, 0.1 mm for the distal deviation 0.05 mm for the vestibular deviation and −0.54 mm for the apical deviation of the implant base, and a deviation of 0.97 mm for the 3D offset, 0.03 mm for the distal deviation, 0.11 mm for the vestibular deviation and −0.53 mm for the apical deviation of the implant tip were found. 

For all values, the deviation for the first left molar was less than for the right first molar, which was no longer implanted with step-by-step instructions (see Table 1).

This shows that, on average, implants are implanted too deep, not vestibular enough and too far distally by implantologists. If we now look at the right and left first molars separately, we see that the left first molar tends to be placed too deep, too far vestibular and not distal enough. The right first molar, on the other hand, also tends to be placed too deep, but insufficiently distal and vestibular. 

In contrast to the group of implantologists, the deviation for the left first molar was not consistently lower than for the right first molar, which was no longer implanted with step-by-step instructions.

The dental students’ implants deviated with a mean value of 3.43° from the planned implant placement. A mean deviation of 1.51 mm for the 3D offset, −0.05 mm for the distal deviation, 0.27 mm for the vestibular deviation and −1.38 mm for the apical deviation of the implant base, and a mean deviation of 1.78 mm for 3D offset, −0.46 mm for the distal deviation, 0.51 mm for the vestibular deviation and −1.36 mm for the apical deviation of the implant tip were found (see Table 2). 

This shows that, on average, the dental students place implants too deep, not vestibular enough and also too far distal. If we now look at the right and left first molars separately, we see that the left first molar tends to be placed too deep, and not vestibular and distal enough (implant base), or too distal (implant tip). The right first molar also tends to be placed too deep, but not vestibular enough and too far distal. 

Analyzing deviations for 3D-planned implant placement between dental students and implantologists, we found almost all values to be significantly different, except the distal deviation of the implant base (*p* = 0.579) and implant tip (*p* = 0.279), as well as vestibular deviation of the implant base (*p* = 0.246) and implant tip (*p* = 0.225) (see Table 3).

**Figure 8:**
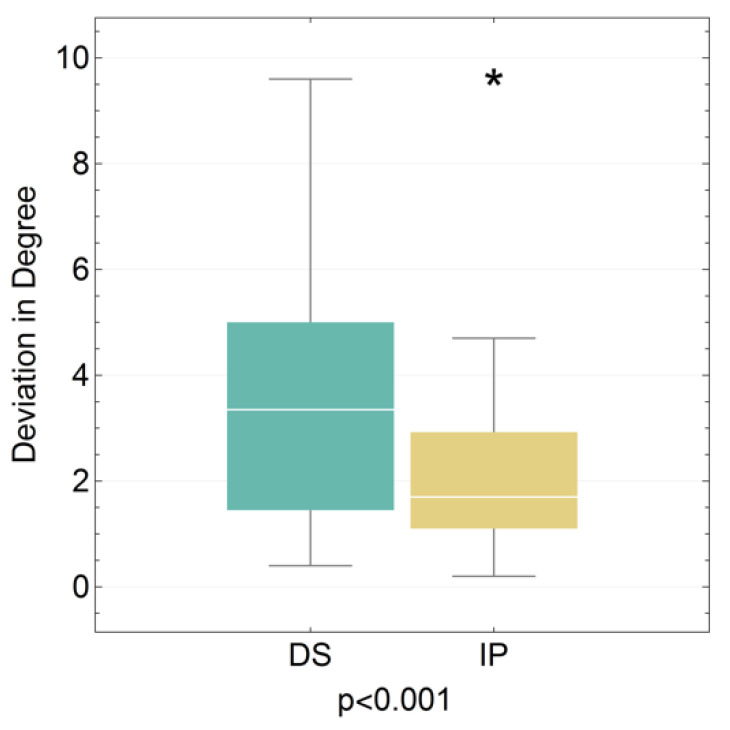
Boxplot for the angular deviation of the dental student (DS) and implantologist (IP) groups (see Table 4). (* extreme values).

In particular, we were able to show that the deviation of the implant angle differs highly significantly between the group of dental students and implantologists (*p* < 0.001). On average, the dental students produced deviations of 3.43° and the implantologists 2.13° from the planned implant placement (Figure 8).

**Figure 9:**
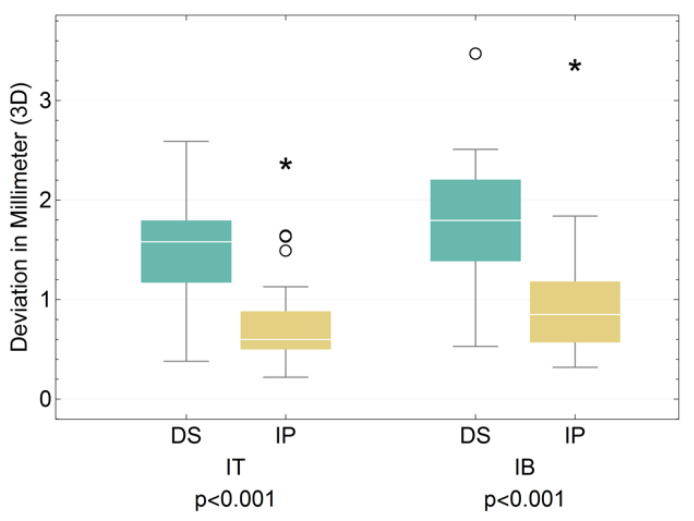
Boxplot for the 3D offset of the dental student (DS) and implantologist (IP) groups at the implant tip (IT) and implant base (IB) (see Table 5). (*: extreme values; O: outliers).

We were also able to show that the 3D offset of the implant also differed highly significantly between the groups of dental students and implantologists (*p* < 0.001). On average, the dental students produced deviations from the planned implant placement by 1.51 mm in the area of the implant base and 1.78 mm in the area of the implant tip, while the implantologists produced deviations of 0.74 mm and 0.97 mm, respectively (Figure 9).

**Figure 10:**
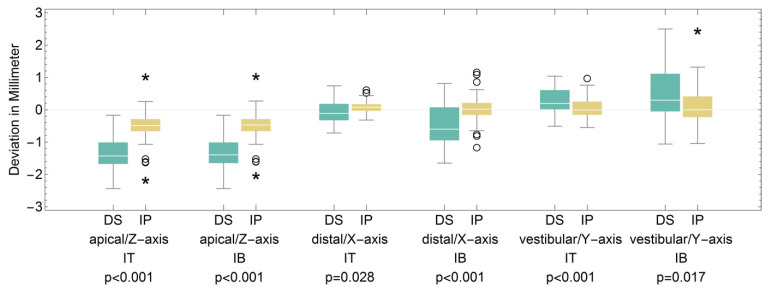
Boxplot for the deviation in X-, Y- and Z-axes of the dental student (DS) and implantologist (IP) groups at the implant tip (IT) and implant base (IB) (see Table 6). (*: extreme values; O: outliers).

Furthermore, we were able to show that both groups also particularly differed highly significantly in the deviations of the implants in the area of the X-, Y- and Z-axes for the implant tip and implant base (Figure 10).

Thus, it appears that correct implant placement in the left first molar region in the mesio-distal direction and in the right first molar region in the vestibulo-oral direction is approximately equally difficult for a majority of the right-handed collective (97.5% of dental students, 90% of implantologists), regardless of the experience of the practitioner.

### 3.2. Cases with Apical/Z-Axis Deviation >2 mm

As a safety distance of 2 mm to the inferior alveolar nerve is programmed into coDiagnostiX™, cases with an implant placement >2 mm in the Z-axis are decisive, as a potential injury to the nerve could have occurred here. This was the case with a total of four implants. Three implants in the dental student group and one implant in the implantologist group were implanted >2 mm lower than planned in the Z-axis. Three of the four implants were in the region of the first left molar, and one implant was in the region of the first right molar. The data show that for all other parameters collected (with the exception of the distal deviation), all maximum deviations combined in these cases (see Table 7 and Table 8).

Of the three cases from the dental student group, all three students stated that they were right-handed. They were 22, 24 and 24 years old. When asked how they would rate their implantological skills in school grades, they indicated: D, E and F. When asked how they would rate their outcome in school grades after implantation, they indicated: B, B and C.

Unfortunately, it is not possible to make a statement on the case from the implantologist group, because the participant did not hand in his completed questionnaire.

## 4. Discussion

Implant dentistry has led to new developments and innovations in dentistry, such as the use of computer-aided planning, and navigation for more precise and effective implant positioning. Overall, the development of dental implantology has changed the way we treat tooth loss. It is now a reliable, proven, and safe option for patients seeking a long-term and permanent solution to replace missing teeth.

Nevertheless, the training of implantologists is still very heterogeneous, especially in Germany [27]. A distinction must be made between postgraduate training programs, with some with several years of clinical specialty or specialist dentist training on the one hand, and curricular, certified part-time further training on the other. Furthermore, implantology specialist dental practices have been formed, which can cover a wide range of implantology cases.

Individual implant systems of various manufacturers are becoming increasingly diverse and differentiated in their application possibilities. While freehand implantation was the gold standard until the beginning of this millennium, 3D-planned and -guided implantations are becoming more and more common, even outside large clinics [1,5,30,31]. 

Therefore, it was important to evaluate the influence of previous implantological knowledge on the accuracy of the placed implants in comparison to preliminary 3D-planned implant placement. This was achieved with a special focus on the size of individual deviations, regarding possible injury to the inferior alveolar nerve or a possible limitation of the prosthetic restoration of the implants due to excessive displacement of the implants in the X- and Y-axes.

The results of this evaluation indicate a high congruence between preoperative planning data and intraoperative results for the implantologists and dental students as well. In accordance with the literature, the deviations in the group of implantologists were in the ranges already described [5]. We were also able to show that the group of dental students, with practically no previous implantological knowledge, differed significantly from the group of implantologists, as expected, but on average they also showed such a small deviation from the 3D planning of the implant positions in the X-, Y- and Z-axes, that a prosthetic restauration of the implants should be possible. Special attention should be paid to the deviation in the Z-axis, and thus a possible injury to the inferior alveolar nerve. On average, dental students implanted too deep, but with a median value of 1.38 mm for the implant base and 1.36 mm for the implant tip. However, since in our setting, the mucosa was not surgically opened and was implanted with a direct vision of the bone, this is less likely in the real patient. Thus, the deviation could either be due to operator error (choosing the wrong drill or tray), or the error could be in the implant system. Since the coDiagnostiX™ planning software considers a safety distance of 2 mm to the inferior alveolar nerve, on average, there is no risk of injury to the nerve even for first-time users in splint-guided implantology. Of 40 dental students, only 2 (5%) implanted deeper than 2 mm compared to the 3D planning, risking injury to the inferior alveolar nerve. Of the 20 implantologists, 1 person (5%) implanted deeper than 2 mm compared to the planning, and thus also risked injury to the inferior alveolar nerve. This result confirms the empirical requirement of a minimum distance of 2 mm to important anatomical structures (inferior alveolar nerve, adjacent tooth) [32].

Nevertheless, for the prevention of injuries to anatomical structures, maximum deviations are the most important values to consider. In particular, the Z-axis is important in regard to possible injuries to the inferior alveolar nerve. In total, we had four cases with a deviation of >2 mm in the Z-axis, three being in the dental student group and one in the implantologists group. All four implants were implanted too deep, with the largest deviation being 2.4 mm. This significant deviation may be explained by the fact that the drilling guide might not be placed correctly, with tension or possible interference in the adaptation of the guide. This has been well described in the literature for “real world” implantation [33] and might also be a reason for using a phantom head, even in implantology. Furthermore, a deviation from the planning can also be characterized by an excessive use of pressure, which can lead to a deformation of the drilling guide and thus a deviation of the implant position [33]. However, placing the implant too deep can also be due to the plastic bone being softer than real bone. It is not possible to say with absolute certainty that the drilling was faulty or whether the fault laid in the insertion of the implant, as here, in contrast to drilling, there is no stop to prevent a too-deep insertion. The same also applies to the final freehand insertion of the dental implant; here, there is increased scope for implant movements, and thus deviation from the actual position is possible [34]. The inappropriate use of guides and a mismatch of the drill with the guide cylinder negatively influences the precision of the implants. In addition, deviations may also be due to preoperative planning. Thus, the implant angulation can be affected by patients’ movement during the scanning process [35]. However, this is also very unlikely in this case, as all cases were based on the same 3D planning.

Other reasons that can lead to a deviation of the implant position, but had no influence in this case due to a training situation without an actual patient, are restricted mouth opening [36], edema of the gingiva after anesthetic infiltration, which can lead to an incorrect fit of the drilling guide [37], the level of the bone crest [5], patient movements during surgery and poor visibility of the surgical area in the posterior region [37].

The highly significant difference in the 3D offset between the two groups in favor of the implantologists is, in our opinion, a sign of the implantologists’ better manual skills in handling the correct positioning of the splint, as well as the simultaneous handling of the splint and the drill. Possible sources of errors mentioned above (except for possible patient movements during the scanning process) can all be attributed to a lack of manual skill and practice. Therefore, a significant deviation between implantologists, who stated that they had already placed >200 implants, and first-time-user dental students is to be expected, which also manifests itself in the clear 3D offset as a marker for manual skills. However, although this was expected, the results show that splint-guided implantology, with its precise planning and strict specification of implant positions, can compensate for the significant differences in manual skill and ensure an adequate implant position, even in first-time users.

The greater deviation of the implants in the area of the implant tip compared to the implant base in the area of the X- and Y-axes described by us is certainly due to the length of the implants, and has been described in a small cohort before [5]. With the increasing implant length, the deviation of the implant tip increased with the smallest angular deviations of the implant base. Our hypothesis of the greater deviation in the implant tip to the implant base has thus been confirmed. We attribute this to a better guidance of the implant at the beginning. Especially with a lack of manual skill and practice, this results in a greater deviation. Further studies with different implant lengths are certainly necessary to describe a significant correlation between the deviation and the implant length. In the meantime, an implant should be planned only as long as necessary and as short as possible.

Of course, the study also has its limitations. On the one hand, the students only placed one implant each, but the implantologists placed two. This was due to the course design and could not be changed due to the number of participants and the equipment with workstations. However, there was no significant difference between the first and second implant placed, especially among the implantologists. The possible training effect with a second inserted implant could therefore not be proven. On the contrary, the precision for the second implant was even lower in the implantologist group. The second implant placed in the jaw was also less precise in the students group. In the authors’ view, this was because the second implant was placed more independently, without the step-by-step instructions as with the first implant. Another limiting factor is the plastic jaw models. Although these anatomically resemble the real jaws of the planning, the internal structure and corresponding resistance during drilling do not correspond to real cortical bone. The gingiva was also only represented by an approx. 1.5 mm thick rubber membrane. It was not possible to open the mucosa here so that the implants were inserted “transgingivally” without visual control on the bone successes. However, transgingival implant placement also an advantage for the study, whereby the “surgeons” completely had to rely on the planning and only adjust the depth of the implant placement slightly. This makes the potential error of the drilling being too deep with the implant placed correctly rather unlikely.

## 5. Conclusions

This study shows that guided implantology provides predictable and reproducible results in dental implantology. Incorrect positioning, injuries to anatomical structures and implant positions that cannot be prosthetically restored can thus be avoided. We were able to show that on average, the deviations from the computer-assisted 3D planning were less than 1 mm for the implantologists, and the students also did not deviate more than 1.78 mm on average from 3D planning. For some parameters of implant positioning, the students even showed a more similar accuracy than the implantologists. This makes guided implantology a beginner-friendly, safe and, above all, reproducible type of dental implantology, and can deliver good-to-very-good results for practitioners at all levels of experience. 

## Figures and Tables

**Table 1:** Comparison of the actual implant placement with the planned implant placement for the implantologist group (IB = implant base, IT = implant tip).

	Angle	3D (IT)	Distal (IT)	Vestibular (IT)	Apical (IT)	3D (IB)	Distal (IB)	Vestibular (IB)	Apical (IB)
Minimum Deviation	0.20	0.22	0	0	−0.03	0.32	0.01	0	−0.02
Maximum Deviation	9.70	2.39	0.64	1.00	−2.14	3.38	1.19	2.48	−2
Median Deviation	1.70	0.60	0.08	−0.01	−0.49	0.85	0.02	0.00	−0.47
Mean Value Deviation	2.13	0.74	0.10	0.05	−0.54	0.97	0.03	0.11	−0.53

**Table 2:** Comparison of the actual implant placement with the planned implant placement for the dental student group (IB = implant base, IT = implant tip).

	Angle	3D (IT)	Distal (IT)	Vestibular (IT)	Apical (IT)	3D (IB)	Distal (IB)	Vestibular (IB)	Apical (IB)
Minimum Deviation	0.40	0.38	0	0	−0.17	0.53	−0.02	−0.04	−0.17
Maximum Deviation	9.60	2.59	0.74	1.04	−2.44	3.49	−1.65	2.50	−2.44
Median Deviation	3.35	1.58	−0.11	0.20	−1.43	1.80	−0.60	0.30	−1.40
Mean Value Deviation	3.43	1.51	−0.05	0.27	−1.38	1.78	−0.46	0.51	−1.36

**Table 3:** Comparison and *p*-values of deviations for 3D-planned implant placement between dental students and implantologists (IB = implant base, IT = implant tip).

	Angle	3D (IT)	Distal (IT)	Vestibular (IT)	Apical (IT)	3D (IB)	Distal (IB)	Vestibular (IB)	Apical (IB)
first left + right molar									
*p*-Value	<0.001	<0.001	0.028	<0.001	<0.001	<0.001	<0.001	0.017	<0.001
first left molar									
*p*-Value	0.055	<0.001	0.579	<0.001	<0.001	<0.001	0.279	0.015	<0.001
first right molar									
*p*-Value	<0.001	<0.001	<0.001	0.246	<0.001	<0.001	<0.001	0.225	<0.001

**Table 4:** Corresponding table for the angular deviation of the planned implant placement for the dental student (DS) and implantologist (IP) groups.

	DS	IP
Median	3.35	1.7
Interquartile Range	3.6	1.8
Minimum	0.4	0.2
Maximum	9.6	9.7

**Table 5:** Corresponding table for the 3D offset of the dental student (DS) and implantologist (IP) groups at the implant tip (IT) and implant base (IB).

	IT DS	IT IP	IB DS	IB IP
Median	1.58	0.6	1.795	0.85
Interquartile Range	0.62	0.38	0.84	0.61
Minimum	0.38	0.22	0.53	0.32
Maximum	2.59	2.39	3.49	3.38

**Table 6:** Corresponding table for the deviation in X-, Y- and Z-axes of the dental student (DS) and implantologist (IP) groups at the implant tip (IT) and implant base (IB).

	Apical/Z-Axis IT DS	Apical/Z-Axis IT IP	Apical/Z-Axis IB DS	Apical/Z-Axis IB IP	Distal/X-Axis IT DS	Distal/X-Axis IT IP	Distal/X-Axis IB DS	Distal/X-Axis IB IP	Vestibular/Y-Axis IT DS	Vestibular/Y-Axis IT IP	Vestibular/Y-Axis IB DS	Vestibular/Y-Axis IB IP
Median	−1.43	−0.49	−1.395	−0.47	−0.11	0.08	−0.6	0.02	0.195	−0.01	0.3	0
Interquartile Range	0.66	0.36	0.64	0.36	0.51	0.19	0.99	0.34	0.58	0.39	0.94	0.62
Minimum	−2.44	−2.14	−2.44	−2	−0.72	−0.32	−1.65	−1.14	−0.51	−0.55	−1.06	−1.04
Maximum	−0.17	1.06	−0.17	1.07	0.74	0.64	0.82	1.19	1.04	1	2.5	2.48

**Table 7:** Implant placements in the dental student group with apical deviations >2 mm (bolded deviations are also the maximum deviation for this group; IB = implant base, IT = implant tip).

	Angle	3D (IT)	Distal (IT)	Vestibular (IT)	Apical (IT)	3D (IB)	Distal (IB)	Vestibular (IB)	Apical (IB)
Case 1 (first left molar)	**9.60**	**2.59**	−0.16	**1.04**	−2.37	**3.49**	−0.97	**2.50**	−2.23
Case 2 (first left molar)	3.00	2.38	0.39	0.18	−2.34	2.34	−0.13	0.17	−2.33
Case 3 (first right molar)	1.70	2.47	0.19	0.29	**−2.44**	2.51	0.11	0.57	**−2.44**

**Table 8:** Implant placements in the implantologist group with apical deviations >2 mm (bolded deviations are also the maximum deviation for this group).

	Angle	3D (IT)	Distal (IT)	Vestibular (IT)	Apical (IT)	3D (IB)	Distal (IB)	Vestibular (IB)	Apical (IB)
Case 1 (first left molar)	**9.70**	**2.39**	−0.32	**1.00**	**−2.14**	**3.38**	−1.14	**2.48**	**−2.00**

## Data Availability

Data are available upon reasonable request.
